# Deep Learning-Based Estimation of Reverberant Environment for Audio Data Augmentation

**DOI:** 10.3390/s22020592

**Published:** 2022-01-13

**Authors:** Deokgyu Yun, Seung Ho Choi

**Affiliations:** 1 Department of Electronic Engineering, Seoul National University of Science and Technology, Seoul 139-743, Korea; deokkyuyun@gmail.com; 2 Department of Electronic and IT Media Engineering, Seoul National University of Science and Technology, Seoul 139-743, Korea

**Keywords:** audio data augmentation, dereverberation, deep learning, room impulse response

## Abstract

This paper proposes an audio data augmentation method based on deep learning in order to improve the performance of dereverberation. Conventionally, audio data are augmented using a room impulse response, which is artificially generated by some methods, such as the image method. The proposed method estimates a reverberation environment model based on a deep neural network that is trained by using clean and recorded audio data as inputs and outputs, respectively. Then, a large amount of a real augmented database is constructed by using the trained reverberation model, and the dereverberation model is trained with the augmented database. The performance of the augmentation model was verified by a log spectral distance and mean square error between the real augmented data and the recorded data. In addition, according to dereverberation experiments, the proposed method showed improved performance compared with the conventional method.

## 1. Introduction

Recently, as deep learning-based research progresses, the need for audio data augmentation is increasing, especially in reverberant environments. Given that the performance of a deep learning-based approach depends on how similar the training data are to the real data and how sufficient the data are for training, research on data augmentation techniques is ongoing. In the area of image processing, many methods have been developed to augment data through scale, translation, and rotation [1,2,3]. Existing data augmentation methods for acoustic data—virtual data generated through time stretching or pitch shifting—are used in the training process [4,5,6]. These methods are for data augmentation through modulation of sound data and not the sound transmission effect, according to specific spatial characteristics. When modeling the transmission process of sound, the characteristics of sound are used. The reverberation environment of a room is estimated by modeling the sound transmission process. The conventional estimation methods use the room impulse response (RIR) [7,8,9] or room transfer function (RTF) [10,11], and convolve RIR and clean sound to create a virtual reverberant sound in a specific space. Among recent studies, there is a study of learning an artificial neural network using the structure of a room and acoustic signals acquired from the room, where RT60, which is an attenuation parameter of the sound pressure level, was estimated and used to construct a reverberant signal [12]. In a similar way, the methods for generating acoustic parameters using deep neural networks (DNNs) have been studied. These methods estimate the RIR using generative adversarial networks (GANs) [13] and DNN-based room acoustic parameter estimation methods [14,15]. These recent methods obtain the output reverberation signal by using the original signal as an input to the linear time-invariant system. On the other hand, in this study, considering that the transmission of an actual acoustic signal has a non-linear characteristic, the non-linear system is modeled using a deep learning technique and the output reverberation signal is obtained by using it.

This study is to obtain a clean signal from a reverberant signal as a preprocessing process for speech applications, such as speech recognition or speech communication, in a reverberant environment. To do this, a large database is required to utilize the deep learning techniques, which are currently showing the best performance. In a real situation, if only limited recorded data is used, the performance of the deep learning model cannot be guaranteed. Therefore, there is a need for a data augmentation technique that uses limited reverberant data to obtain more realistic data.

The sound generated through RIR, when a person hears it, is similar to the sound actually recorded, but there is a problem in applying it as training data to a data-driven model such as a deep neural network because the distance between the generated and recorded sound can be considerable. To solve this problem, in this paper, we try to train a deep neural network model that uses some of the recorded sounds to make the augmented data more similar to the recorded data than the sounds generated by the RIR. The proposed method estimates a reverberation environment model based on a deep neural network that is trained by using clean and recorded audio data as inputs and outputs, respectively.

## 2. Conventional Reverberant Environment Estimation Method

Previously, an artificial RIR was used for the modeling and analysis of sound transmission processes in a room, and the reverberant signal was generated by the convolution of clean data with this RIR [7]. A direct sound reaches a specific location with the loudest sound, and, after a delay time, the sound reflected from the wall, ceiling, or floor arrives with a reduced sound pressure.

**Figure 1:**
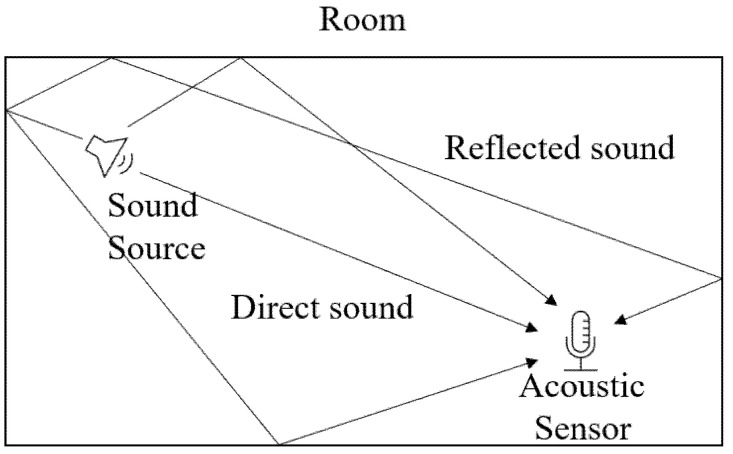
Example of acoustic transmission in a room.

As shown in Figure 1, the direct sound arrives the fastest and loudest, and then the reflected sounds arrive with a time difference. The RIR h(n) is generated through this model, and artificial reverberant data $\hat{y}\left(n\right)$ are generated by convolving the RIR with the clean sound x(n), as shown in the following equation,

(1) $$\hat{y}\left(n\right)=\sum_{m=0}^{L-1}h\left(m\right)x\left(n-m\right)$$

If the clean sound is filtered through this RIR, it can have a similar effect as hearing the sound in the room. Conventionally, the RIR is generated using a wave equation or an image method, which is a knowledge-based method [7]. It can be used to give spatial effects for human hearing in a room. However, it may be inappropriate because there can be a large difference between the augmented data by the RIR and the actual recorded data because the process of acoustic transmission is non-linear. Therefore, this research work aims to generate data that are more similar to the actual recorded data than that generated by the existing method.

## 3. Proposed Reverberant Data Augmentation Method

**Figure 2:**
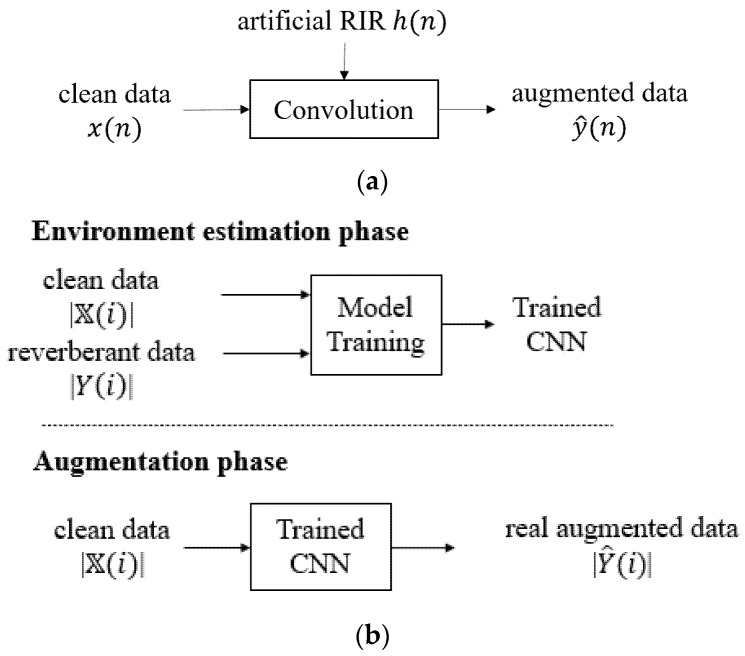
Block diagram of (**a**) conventional method and (**b**) proposed method.

The proposed augmentation method is to generate reverberant data through a convolutional neural network (CNN) [16]. The difference between the proposed and existing methods of data augmentation is shown in Figure 2. Conventionally, an artificial RIR is estimated using the information of the room structure, and the reverberant signal is generated by the convolution of clean data with the RIR as in Figure 2a. However, the proposed method first trains a CNN model using both a clean signal and recorded reverberant signal at the environment estimation phase. Then, a large amount of real augmented data is constructed with the trained CNN by using clean data as inputs. In Figure 2b, the data are a feature vector of an audio signal, which is the short-time magnitude spectrum. When composing input data, adjacent frames are input together to account for reverberation components. The inputs for model training are both the magnitude spectra of the current and adjacent frames, |X(i)| as in Equation (2), and the magnitude spectrum of the reverberant signal, |Y(i)|.

(2) $$\left|X\left(i\right)\right|=\{\left|\vec{X}\left(i-l\right)\right|,\ldots ,\left|\vec{X}\left(i\right)\right|,\ldots ,\left|\vec{X}\left(i+l\right)\right|\}$$

As well, the phase of the $\left|\hat{Y}\left(i\right)\right|$, which is the output of CNN, uses the phase of the clean sound.

**Figure 3:**
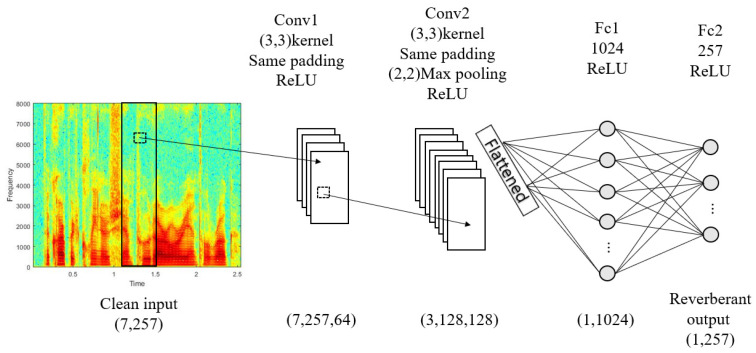
Block diagram of reverberant environment estimation using CNN model.

The overall structure of the CNN model is shown in Figure 3, where Conv and Fc represent a convolution layer and a fully connected layer, respectively.

Furthermore, to verify that the proposed data augmentation method is helpful in constructing data for dereverberation, we trained a deep neural network for dereverberation with the data generated by each method. The dereverberation method is learning the ideal ratio mask (IRM) [17] of the clean spectrum compared to the reverberant spectrum. In the IRM method, a spectrum of a clean signal is obtained by covering the reverberant input spectrum with a mask of an appropriate ratio. After that, the dereverberated signal is obtained through an inverse short-time Fourier transform. As with the reverberant environment estimation method, the phase of the output data uses that of the reverberant sound.

## 4. Experiments and Results

**Figure 4:**
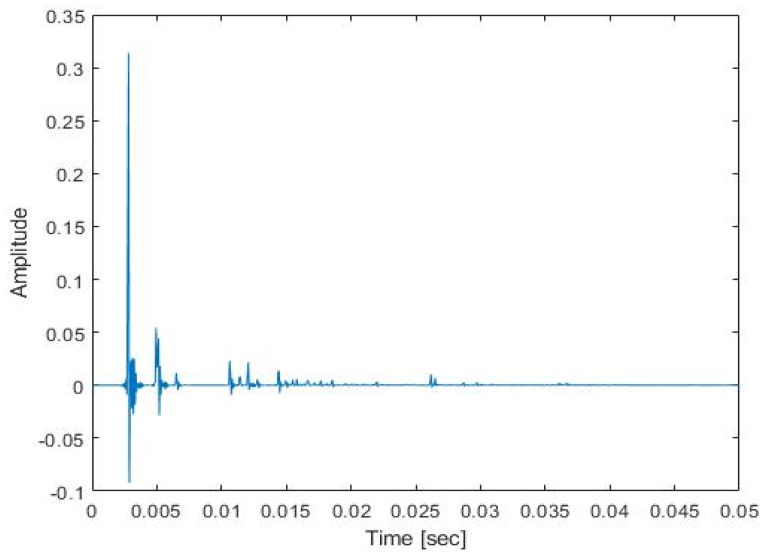
The room impulse response of the experiments.

**Figure 5:**
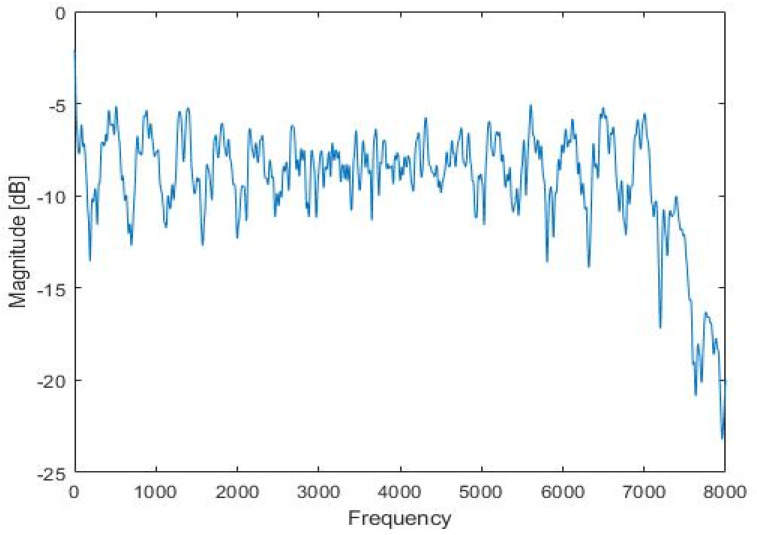
The room transfer function of the experiments.

The proposed method needs acoustic data recorded in a reverberant environment. For the CNN-based environment estimation, the training database is constructed by recording the TIMIT speech database [18] played in an indoor space of 4250, 3300, and 2700 mm. The microphone and speaker are positioned at 1700, 2000, and 800 mm and 1700, 400, and 600 mm. Both the conventional method and the proposed method played and recorded at the same position. Figure 4 and Figure 5 show the RIR and RTF obtained through the existing method in this space. The TIMIT database consists of 4620 and 1680 sentences for training and testing, respectively. 1000 sentences of the 4620 sentences are used for the training of the reverberant environment estimation model and the rest of the 3620 sentences are later used for the training of the dereverberation model.

### 4.1. Result of the Proposed Data Augmentation Method

**Figure 6:**
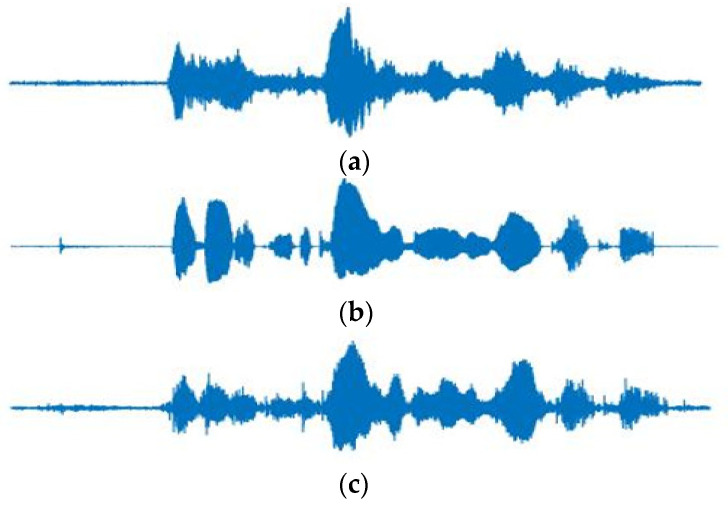
Waveform examples of (**a**) the recorded signal, (**b**) the signal artificially generated by RIR, and (**c**) the signal generated by the proposed method.

**Figure 7:**
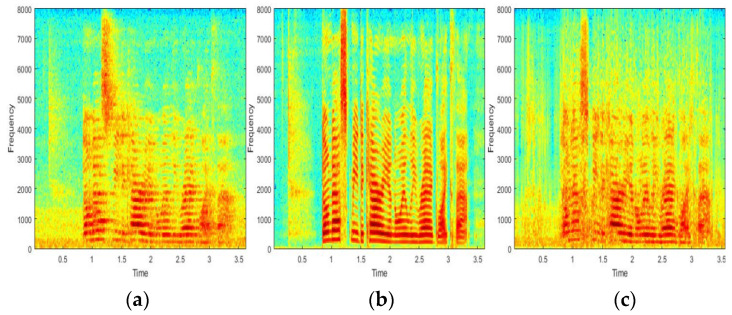
Spectrogram examples of (**a**) the recorded signal, (**b**) the signal artificially generated by RIR, and (**c**) the signal generated by the proposed method.

The speech signal is divided by a frame size of 20 ms and multiplied by the Hanning window with a 50% overlap [19] to obtain the spectral magnitude. The CNN model in Figure 3 generates the output of the single spectrum vector when seven consecutive frame vectors of clean signals are the inputs. Therefore, with a sampling rate of 16 kHz, the input and output sizes are 7257 and 1257, respectively. Each convolution layer extracts features for a given input and generates an output through a fully connected layer. The ReLU [20] was used for the activation function of each layer and the Adam [21] was used as the optimization function. Furthermore, we stopped the training when the accuracy and loss functions converge. Figure 6 and Figure 7 show waveform and spectrogram examples for the comparison. As shown in the figures, the ones obtained by the proposed method are similar in that they have less distance to the recorded ones than those obtained by the existing method. The comparison results are given in Table 1.

For the verification of the augmentation performance, the root mean square error (RMSE) and log spectral distance (LSD)—as in Equations (3) and (4)—between the data augmented by each method and the actual recorded data are given in Table 1. The actual recorded data consist of 1680 sentences. The proposed method showed a better performance than the conventional methods in both the RMSE and LSD.

(3) $$\mathrm{LSD}=\frac{1}{M}\sum_{i=1}^{M}\sqrt{\frac{1}{K}\sum_{k=1}^{K}10\log{\left(\frac{{\left|Y\left(i,k\right)\right|}^{2}}{{\left|X\left(i,k\right)\right|}^{2}}\right)}^{2}}$$

(4) $$\mathrm{RMSE}=\sqrt{\frac{1}{N}\sum_{n=1}^{N}{\left(x\left(n\right)-y\left(n\right)\right)}^{2}}$$

where i and k are frame index and frequency bin index, respectively. Moreover, for the performance evaluation of dereverberation, we used the perceptual evaluation of speech quality (PESQ) [22], which is the most widely known metric for measuring the quality of the speech signal [23]. In Table 1, the proposed method presents a 2.93 dB LSD and 0.48 PESQ improvement.

### 4.2. Result of Dereverberation

**Figure 8:**
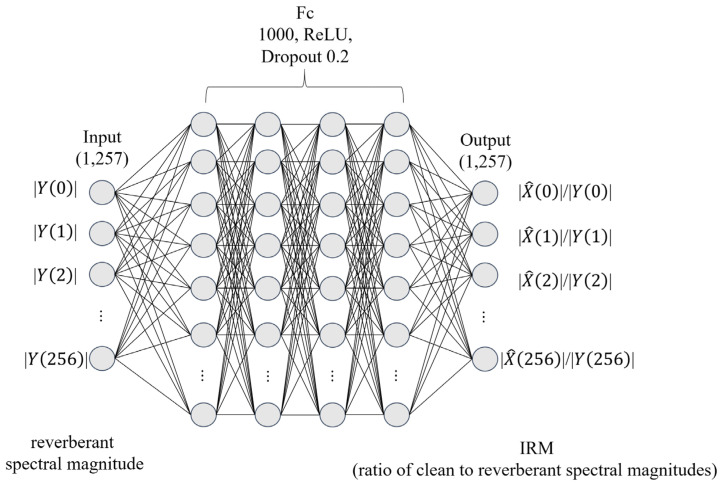
Block diagram of dereverberation model.

**Figure 9:**
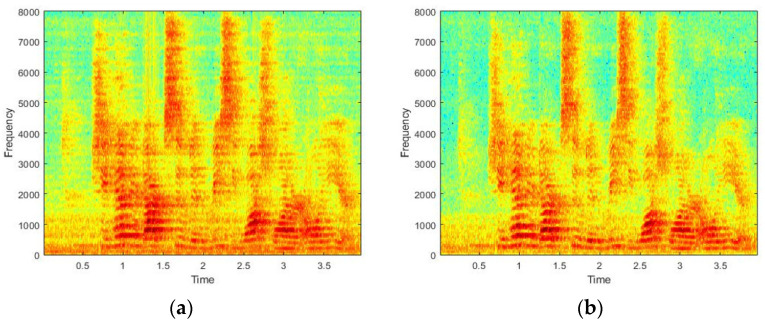
Spectrogram examples of dereverberated signal by (**a**) the RIR method and (**b**) the proposed method.

The speech data to be tested were 1680 sentences, and 3620 sentences were used as the training data. These 3620 sentences were not used to estimate the reverberation environment and were generated by the proposed method rather than the actual recorded speech. Figure 8 is the structure of the IRM model for dereverberation, and Figure 9 is the spectrogram examples of the dereverberated signal from each method. As shown in the figure, the proposed method obtains a cleaner signal than the existing method. The results of comparing the dereverberation performances are shown in Table 2. As a result, the proposed data augmentation method performed better than that from the RIR.

## 5. Discussion

Conventional methods, such as the RIR method, assume that the acoustic reverberation signal is the output of a linear system. However, the actual transmission of the acoustic signal has a non-linear characteristic. This proposed method has novelty in modeling non-linear characteristics by deep learning techniques. Through this study and the experiments, it was found that the proposed method can generate augmented audio data that are more realistic than the existing data augmentation technique. Moreover, the large amount of augmented data was successfully used to train the deep learning model for dereverberation. The proposed method can be adopted as a preprocessing tool for speech recognition or speech communication, especially in a heavy reverberant environment such as a cafe or restaurant. In addition, this method has the advantage that a developer without expertise in acoustics and architecture can effectively augment large amounts of data. For future research, theoretical and experimental studies are needed to model the entire acoustic environment by considering not only the reverberant signal of the target signal but also the background noise.

## 6. Conclusions

In this paper, we presented a novel audio data augmentation method based on deep learning in order to improve the performance of dereverberation. The reverberation environment is estimated by training a convolutional neural network using clean and recorded data. In this way, it was possible to generate data more similar to the actual recorded sound than the conventional RIR method, and it was verified through RMSE and LSD. In addition, we tested the effectiveness of the proposed augmentation method for dereverberation by using the large amount of augmented data. As a result of the experiment, the proposed method showed an improved performance compared with the conventional method. Therefore, the proposed method can be adopted in a preprocessing step in order to enhance the performance of speech applications, such as speech recognition or speech communication.

## Figures and Tables

**Table 1:** LSD and RMSE between recorded and generated signals.

Distance	RMSE	LSD [dB]	PESQ
RIR	0.1625	14.08	1.44
Proposed	0.1562	11.15	1.92

**Table 2:** Performance of dereverberation.

Distance	RMSE	LSD [dB]	PESQ
RIR	0.1409	12.27	1.89
Proposed	0.1361	11.71	1.92
